# Network pharmacology and molecular docking study for biological pathway detection of cytotoxicity of the yellow jasmine flowers

**DOI:** 10.1186/s12906-023-03987-w

**Published:** 2023-05-20

**Authors:** Seham S. El-Hawary, Marzough A Albalawi, Ayat O. S. Montasser, Shaimaa R. Ahmed, Sumera Qasim, Ali A. Shati, Mohammad Y. Alfaifi, Serag Eldin I. Elbehairi, Omnia F. Hassan, Abdelfattah A. Sadakah, Fatma A. Mokhtar

**Affiliations:** 1 Department of Pharmacognosy, Faculty of Pharmacy, Cairo University, Kasr El-Aini Street, Cairo, Egypt; 2 Department of Chemistry, Alwajh College, University of Tabuk, Tabuk, 71491 Saudi Arabia; 3 National Organization for Drug Control (NODCAR), Cairo, Egypt; 4 Department of Pharmacognosy, College of Pharmacy, Jouf University, Sakaka, Aljouf 72341 Saudi Arabia; 5 Department of Pharmacology, College of Pharmacy, Jouf University, Sakaka, Aljouf 72341 Saudi Arabia; 6 King Khalid University, Faculty of Science, Biology Department, Abha, 9004 Saudi Arabia; 7 Department of Pharmacology and Toxicology, Faculty of Pharmacy, MSA University, 6th of October City, Egypt; 8 Oral and Maxillofacial Surgery Department, Faculty of Dentistry, Tanta University, Tanta, Egypt; 9 Oral and Maxillofacial Surgery Department, Faculty of Dentistry, ALsalam University, Kafr Alzayat, Al Gharbia, Egypt; 10 Department of Pharmacognosy, Faculty of Pharmacy, Al Salam University, Kafr Alzayat, Al Gharbia, Egypt; 11 Department of pharmacognosy, Faculty of Pharmacy, El Saleheya El Gadida University, El Saleheya El Gadida 44813, Sharkia, Egypt

**Keywords:** Apoptosis, LC/MS/MS, *Jasminum humile*, MCF-7, Oleaceae, Network pharmacology, Molecular docking

## Abstract

**Background:**

The yellow jasmine flower (*Jasminum humile* L.) is a fragrant plant belonging to the Oleaceae family with promising phytoconstituents and interesting medicinal uses. The purpose of this study was to characterize the plant metabolome to identify the potential bioactive agents with cytotoxic effects and the underlying mechanism of cytotoxic activity.

**Methods:**

First, HPLC–PDA-MS/MS was used to identify the potential bioactive compounds in the flowers. Furthermore, we assessed the cytotoxic activity of the flower extract against breast cancer (MCF-7) cell line using MTT assay followed by the cell cycle, DNA-flow cytometry, and Annexin V-FITC analyses alongside the effect on reactive oxygen species (ROS). Finally, Network pharmacology followed by a molecular docking study was performed to predict the pathways involved in anti-breast cancer activity.

**Results:**

HPLC–PDA-MS/MS tentatively identified 33 compounds, mainly secoiridoids. *J. humile* extract showed a cytotoxic effect on MCF-7 breast cancer cell line with IC_50_ value of 9.3 ± 1.2 µg/mL. Studying the apoptotic effect of J. humile extract revealed that it disrupts G2/M phase in the cell cycle, increases the percentage of early and late apoptosis in Annexin V-FTIC, and affects the oxidative stress markers (CAT, SOD, and GSH-R). Network analysis revealed that out of 33 compounds, 24 displayed interaction with 52 human target genes. Relationship between compounds, target genes, and pathways revealed that *J. humile* exerts its effect on breast cancer by altering, Estrogen signaling pathway, HER2, and EGFR overexpression. To further verify the results of network pharmacology, molecular docking was performed with the five key compounds and the topmost target, EGFR. The results of molecular docking were consistent with those of network pharmacology.

**Conclusion:**

Our findings suggest that *J. humile* suppresses breast cancer proliferation and induces cell cycle arrest and apoptosis partly by EGFR signaling pathway, highlighting *J. humile* as a potential therapeutic candidate against breast cancer.

**Supplementary Information:**

The online version contains supplementary material available at 10.1186/s12906-023-03987-w.

## Background

Cancers are a group of diseases characterized by uncontrolled and unrestricted cell proliferation. Cancers may continue to progress, resulting in premature death, as they alter cell dynamics, cell growth, survival, and differentiation, causing them to become invasive. [1]. Despite the presence of numerous powerful anticancer agents, the majority of these agents have severe side effects. Some cancers respond well to surgery, while others benefit more from medications such as chemotherapy. Inducing apoptosis is considered a powerful cancer-fighting strategy [2]. Apoptosis can be induced by signals from inside the cell, such as genotoxic stress, or by extrinsic signals, such as the binding of ligands to cell surface death receptors [2]. Apoptosis, or programmed cell death, is mediated by an intracellular proteolytic cascade and is finely regulated at the gene level to produce the orderly addition to efficient elimination of impaired cells in a tremendously controlled manner without concomitant inflammatory reactions [3–5]. Although the accumulation of free radicals inside the body increases the susceptibility of cancer development, tumor cells are affected by the variations in reactive oxygen species (ROS) levels [6] as they are capable of eliciting oxidative stress. As a result of the release of pro-apoptotic proteins, macromolecular and organelle damage occurs, resulting in apoptosis and activation of antioxidant pathways in surviving cells.

Italian jasmine *Jasminum humile* L. (*J. humile*) is a fragrant plant belonging to the Oleaceae family, known as Italian jasmine or yellow jasmine. It is a small branched shrub with yellow fragrant flowers [7], its fragrant oil is used in the perfume industry and the yellow dye from the flowers and roots was used as a natural dye for solar cells [8]. Extracts of several species of the genus *Jasminum* revealed different pharmacological activities as anticancer [9–11], antioxidant [10, 12, 13], chemo-preventive, antibacterial [14, 15], analgesic [16], gastroprotective [17], and anti-inflammatory activities [10].

The present study explored the cytotoxic activity of a methanol extract of *J. humile* by MTT assay against breast cancer (MCF-7) cell line, combined with high-performance liquid chromatography/photodiode array coupled to mass spectroscopy-mass spectroscopy (HPLC–PDA-MS/MS) analysis to evaluate the role of the plant constituents in the plant cytotoxic activity. GO biological process analysis and KEGG pathway enrichment analysis were used to investigate the potential mechanisms underlying *J. humile*'s anti-breast cancer activity. The top-hit compounds were molecular docked against the top-hit molecular target relevant to breast cancer identified in the constructed networks to learn more about how these compounds interact with the active regions of the target proteins. Such a strategy could help researchers better understand *J. humile*'s molecular mechanism of action in breast cancer treatment.

## Materials and methods

### Plant material and extraction

*Jasminum humile* L. in the flowering stage was collected on 20^th^ May 2019 from EL-Keram farms, Moderayat al-Tahrir, El-Behaira government, Egypt. It was taxonomically identified by Dr. Mohammed El Gebaly (Consultant botanist-Orman Garden) and a voucher specimen (3.10.16.1) was kept in the herbarium of Faculty of Pharmacy, Cairo University. The plant flowers were collected, washed, and shadow dried. The flowers were then pulverized to a coarse powder (2–3 mm) with an electric blender before extraction. Pulverized flower (50 g) was extracted in a percolator with 80% methanol for 24 h at room temperature. Three washes with 100 mL of fresh solvent with a hold time of 5 h were carried out until the last extract was colorless. The combined extract was filtered Whatman No.1 filter paper and was further concentrated under vacuum using a rotary evaporator at 45 °C to yield 10.5 g dry extract. The crude solid extract was labeled and stored under cold conditions before analysis.

### HPLC–PDA-MS/MS

A Thermo Finnigan LC system was used for the HPLC–PDA-MS/MS analysis of *J. humile* extract (Thermo Electron Corporation, Austin, TX, USA). The instrument utilized was a Zorbax Eclipse XDB-C18, Rapid resolution, 4.6 150 mm, with a 3.5 µm column (Agilent, Santa Clara, CA, USA). Acetonitrile concentration was raised from 5 to 30% in 60 min at a flow rate of 1 mL/min and a 1:1 split before the ESI source using gradient elution with water and acetonitrile (ACN), each containing 0.1% formic acid. Thermo Quest ESI source-equipped LCQ-Duo ion trap was utilized for MS analysis. The system was managed by Xcalibur software (XcaliburTM 2.0.7, Thermo Scientific, Waltham, MA, USA). The MS operating settings were employed in the negative mode [18].

### Biological evaluation

#### MTT assay

MCF-7 cells were maintained in RPMI-1640 medium supplemented with 10% FBS, glutamine (2 raM), penicillin (100 units/mL), and streptomycin (100 µg/mL). The cells were cultured at 37 °C in a humidified 5% CO_2_ incubator. The extract of *J. humile* flower was tested for in vitro cytotoxicity, using MCF-7 cells by 3-(4,5-dimethylthiazol-2-yl)-2,5-diphenyltetrazolium bromide (MTT) assay. One hundred µL of (RMPI 1640) media was loaded into each of the 96-well plates (triplicate). The final volume for each well was 100 µL. The cultured MCF-7 cells were pooled in a 50 mL vial. Then, the cells were plated at a density of 1 × 10^6^ cells/mL cells/well (100 µL) into 96-well microtiter plates. Each sample was replicated 3 times and the cells were incubated at 37 °C in a humidified 5% CO2 incubator for 24 h. After the incubation period, MTT (20 µL of 5 mg/mL) was added to each well and the cells were incubated for another 2–4 h until purple precipitates were clearly visible under a microscope. Flowingly, the medium together with MTT (190 µL) were aspirated off the wells, DMSO (100 µL) was added, and shake the plates for 5 min. Measure the absorbance spectrophotometry at 540 nm in a microtiter plate reader and the percentage cell viability was calculated manually using the formula:

$$\%\;\mathrm{cell}\;\mathrm{viability}\:=\:(\mathrm{average}\;\mathrm{abs}\;\mathrm{duplicate}\;\mathrm{drug}\;\mathrm{wells}\;/\mathrm{average}\;\mathrm{abs}\;\mathrm{control}\;\mathrm{wells})\:\times\:100$$

A dose–response curve was plotted to enable the calculation of the concentrations that kill 50% of the MCF-7 cells (IC_50_) compared to the standard drug, etoposide, and the effect of the extract on normal keratinocyte cells (HaCaT).

#### DNA-flow cytometry analysis

The influence of *J. humile* extract (5, 10, and 20 µg/mL) on the cell cycle distribution of MCF-7 cell line was evaluated using the CycleTEST™ PLUS DNA Reagent Kit (Becton Dickinson Immunocytometry Systems, San Jose, CA) according to the manufacture instructions. PBMC cells served as control cells with known DNA content for determining the DNA Index (DI) of the inspected samples. Propodium iodide (PI) was used as a DNA-binding dye before running on the DNA cytometer [19]. CELLQUEST software was used for analyzing Cell-cycle distribution.

#### Annexin V-FITC apoptosis assay

Annexin V-FITC/DAPI assay (Cayman Chemical, Ann Arbor, MI) was used to analyze apoptotic cells. After culturing MCF-7 cells into a monolayer, they were treated with *J. humile* extract at 5, 10, and 20 µg/mL. Cells were subsequently collected through trypsinization, double washed in phosphate buffer saline (PBS) followed by the binding buffer. Afterward, cells were re-suspended in 100 µL of binding buffer with the addition of 5 µL of FITC-Annexin V (Becton Dickinson BD PharmingenTM, Heidelberg, Germany) followed by a 30 min. incubation period at 4 °C. Cells were then washed in binding buffer and re-suspended in 150 µL of binding buffer with the addition of 1 µL of DAPI (1 µg/µL in PBS) (Invitrogen, Life Technologies, Darmstadt, Germany). The flow cytometer BD FACS Canto II (BD Biosciences, San Jose, CA) was used to analyze the cells and the results were deduced with FlowJo7.6.4 software (Tree Star, FlowJo LLC, Ashland, OR) [20].

#### Oxidative stress parameters

Spectrophotometric assessment of Superoxide dismutase (SOD) activity at 560 nm following the method of [21] was done based on inhibition of nitro blue tetrazolium- NADH and phenazinemethosulphate (PMS)-mediated formazan formation. Spectrophotometric assessment of Catalase (CAT) was done using the method of [21]. The assay measures the decomposition of H_2_O_2_ by CAT at 240 nm. Glutathione reductase (GSH-R) was assessed spectrophotometrically [21] based on the reduction of oxidized glutathione by GSH-R with the help of NADPH. Afterward, the thiol group of reduced glutathione reacts with the chromogen to yield a colored complex measured at 405 nm.

### Network Pharmacology

#### Target Genes Associated with Breast cancer and Selected Compounds

Binding DB (https://www.bindingdb.org/bind/index.jsp) was used using the "homo sapiens" setting to predict target genes for selected compounds based on SMILES. The "minimum needed interaction score" was set to "high confidence (0.700)" during Binding DB prediction. The public database DisGeNET (http://www.disgenet.org/) was used to identify disease-related target genes [22].

#### Interactions between Compounds and Overlapping Genes: Network Construction

Cytoscape ver. 3.9.1 (https://cytoscape.org/) was used to construct, display, and analyze the network of interactions based on the Binding DB prediction results for constituents and overlapping genes. Nodes in the network indicate bioactive components and genes, while edges show interactions between compounds and genes. Anti-breast cancer components and hub genes were identified by analyzing the network's topological structure and setting the "Degree value" of compounds or genes. A compound's or a gene's degree value represents how many phytoconstituents or genes are present in a network. The therapeutic effects of compounds are enhanced if the compounds target more disease-inducing genes [22].

#### Building a Protein–Protein Interaction Network

An online database called STRING (https://string-db.org/) was used to gather information on protein–protein interactions between the target proteins of selected *J.humile* components (PPI). The website calculated a score for each protein's mutual information. The stronger the contact between the two target proteins, the higher the score. Since high-confidence data > 0.7 were used to ensure accuracy and reliability, the study was considered reliable. The obtained protein interaction data was imported into the Cytoscape 3.9.1 application to generate a PPI protein interaction network. The CytoHubba plug-in was employed for the identification of Hub genes [23]. The cyto hubba plg-in has 12 topological features among which we used the “Degree” parameters to screen out the top-ranked proteins according to their degree value of interaction.

#### Target Protein Gene Ontology and KEGG Enrichment Analysis

It was found that proteins that interact with the active components of *J.humile* play a role in gene function and signaling pathways by using the database for annotation, visualization, and integrated discovery (David) v 6.8 to evaluate gene function and KEGG pathway enrichment, respectively [24]. The functional enrichment database DAVID, which is accessible online, aids researchers in understanding the bioactivity of a large number of genes. In the current study, p-value ≤ 0.01 was chosen, and the top 10 KEGG pathways and GO enrichments were picked for further investigation. Cellular components (CC), molecular functions, biological processes, and pathways were all included in the list of target proteins. KEGG pathway enrichment results were used to decipher the possible molecular mechanisms of *J.humile* against breast cancer. KEGG pathway bubble charts made with SRPLOT (http://bioinformatics.com.cn/) were of particular interest.

### Molecular docking

The current study used the MOE-Dock from Chemical Computing Group Inc. to carry out computational experiments. The crystal structures of EGFR were obtained using the Protein Data Bank (PDB id 4HJ0). After editing the crystal structures to remove the water molecules and add hydrogen atoms to the protein, MOPAC 7.0 was used to minimize the energy. Then, the alpha spheres produced were used to create dummy atoms by identifying the active site. In docked regions, RMSD values of less than 1.3 were clustered together. The docked complex in the lowest energy-minimized stance was chosen for further investigation. Ten different conformations were carefully chosen. The MMFF94x force field energy computation was then used for the resulting docked complex model to determine the energy parameters and estimate the docked interactions at the active site [25].

### Statistical analysis

All data are displayed as the mean ± S.D of three separate determinations. The arithmetic mean and the standard deviation (SD) of all assessments were calculated by the software program Microsoft Excel 2007. Statistical analysis was performed using GraphPad Prism version 5.01. To establish the statistical significance in relation to the reference standard, a one-way ANOVA was employed, followed by a Tukey-posthoc test.

## Results

### HPLC–PDA-MS/MS metabolites profiling

**Fig. 1:**
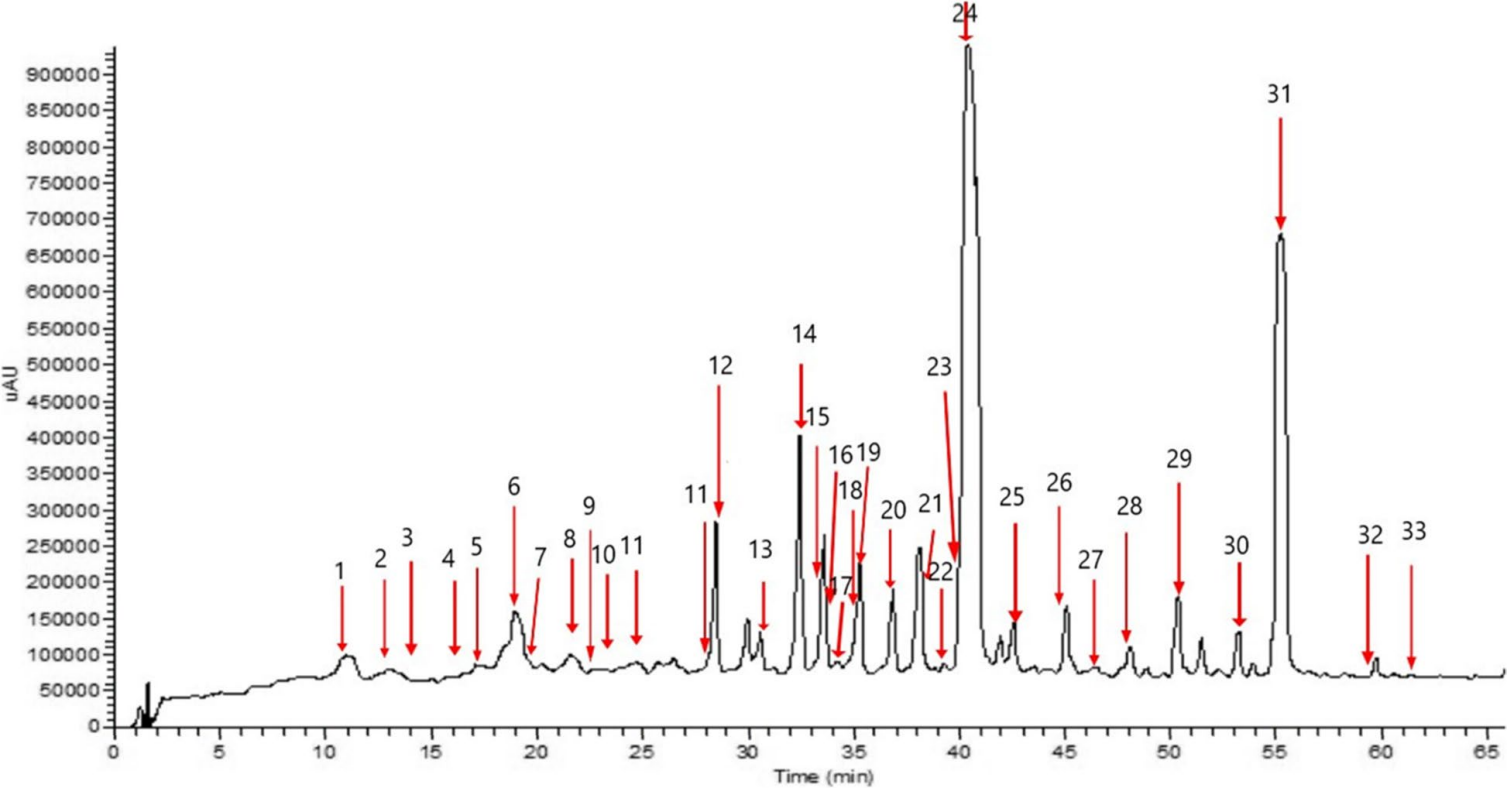
HPLC–PDA-MS/MS chromatogram of *J. humile* flower extract

**Fig. 2:**
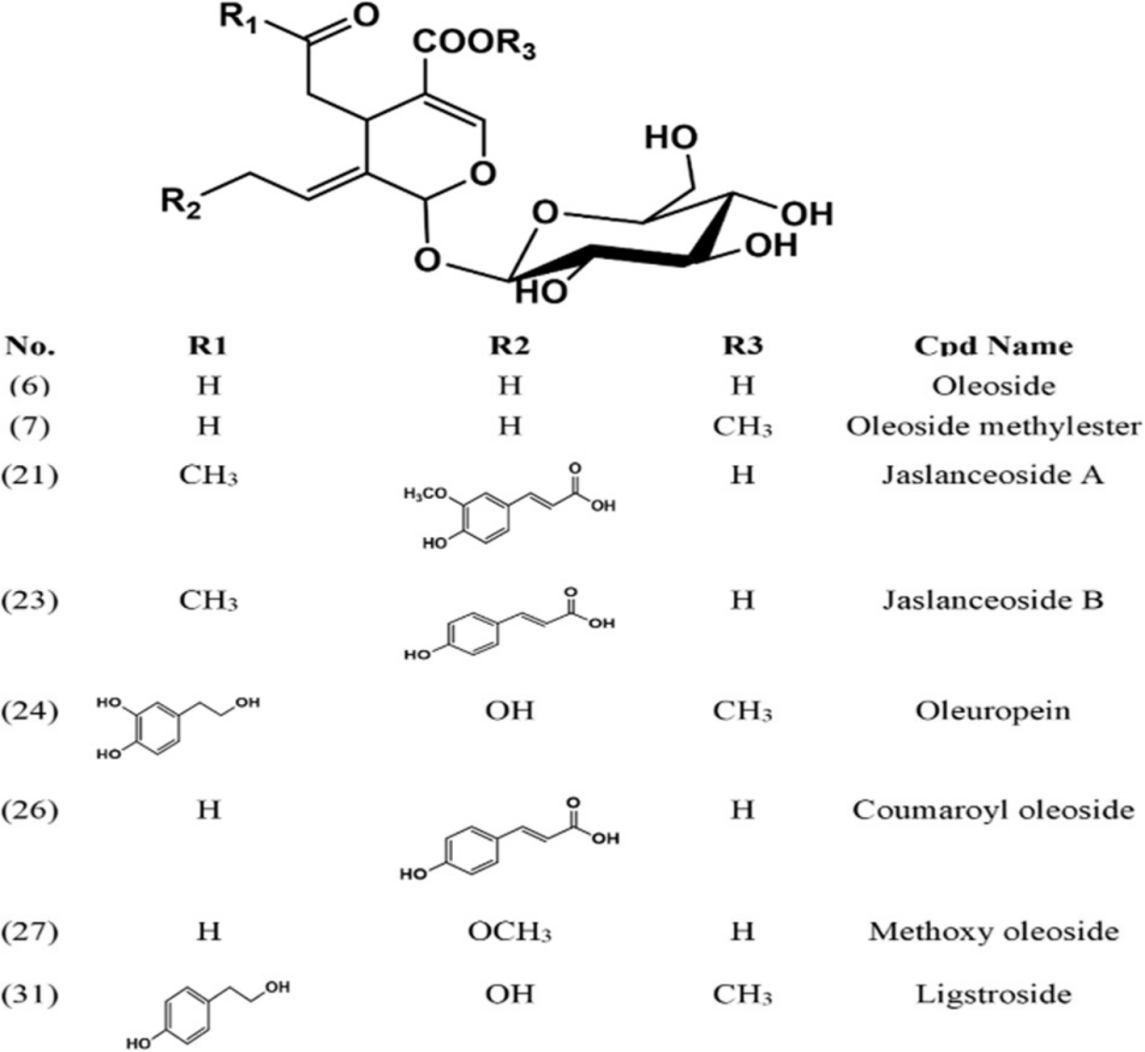
Some secoiridoids identified tentatively from *J. humile* flower

The aqueous methanolic extract of *J. humile* flower was analyzed by HPLC–PDA-MS/MS to characterize its metabolome and identify potential bioactive agents. The analysis tentatively identified 33 compounds (Fig. 1 & Table 1) belonging to various classes [26–41]. The major phytochemical classes are secoiridoids, phenylethanoids, Phenolic acids, flavonoids, and lignans. The major compounds were identified as oleoside derivatives (Fig. 2) and kaempferol glucosides.

**Table 1:** Tentative identification of the chemical composition of aqueous methanolic extract of *J. humile* flower by HPLC–PDA-MS/MS

No	Compound Name	R_t_ (min)	[M-H]^−^	MS/MS	UV	Ref
**Simple phenols**						
1	^B*^ Tyrosol glucoside	11.35	299	137	272	[26]
2	^B*^ Tyrosol	12.70	137	119,93	272	[26]
3	^B*^ Chlorogenic acid	13.82	353	191, 179	281	[27]
4	Ethyl cinnamate	16.44	175	147	284	[28]
9	Sinapic acid glucoside	22.71	385	223,179,161	288	[27]
10	Rosmarimic acid glucoside	23.65	521	359,294,179	271,289	[27]
11	^B*^ Coumaric acid	24.75	163	119	280	[27]
**Lignans**						
8	^B*^ Cycloolivil glucoside	21.19	537	375,327,195	n.d	[29]
14	^B*^ Cycloolivil	31.23	375	345, 195	n.d	[29]
**Flavonoids**						
12	^B*^ Quercetin 3,7-diglucoside	28.18	625	463, 301	255, 350	[30]
13	Quercetin xylosyl glucoside	29.79	595	463, 301	255, 360	[30]
15	^A*^ Quercetin deoxyglucoside glucoside	32.44	609	463, 301	256, 358	[30]
16	^B*^ Quercetin 3-glucoside	33.6	463	301, 179	256, 359	[30]
17	Isorhamnetin-3-O- diglucoside	34.09	639	477, 315	255, 331	[31]
18	^B*^ Kaempferol deoxyhexosyl glucoside	34.95	593, 1187	447, 285	255, 347	[30]
19	Kaempferol 3-xyloside-7-glucoside	35.21	579	447, 285	256, 338	[30]
20	^B*^ kaempferol-3-O-D-glucoside	36.78	447	285	255, 343	[30]
22	Isorhamnetin-3-O-glucoside	39.33	477	315, 194	255, 331	[31]
30	Caffeoyl kaempferol pentoside	53.99	579	417, 285	267,284,341	[32]
**Secoiridoids**						
5	Multiflorin glucoside	17.74	401	239	238	[33]
6	^A*^ Oleoside methyl ester	19.15	403	241, 223	233	[34]
7	^B*^ Oleoside	19.66	389	345, 241,209	230	[35]
21	^B*^ Jaslanceoside A	38.67	595	433,415,389	222, 288	[36]
23	^B*^ Jaslanceoside B	40.25	565	403,221,179	229,290	[36]
24	^A*^ Oleuropein	42.76	539	377,307	231,277	[26]
25	Isojaspolyanoside B glucoside	40.88	967	805, 403	234, 298	[37]
26	^B*^Coumaroyl oleoside	45.08	535	389,345,163	230,286	[38]
27	^B*^ Methoxy oleoside	46.61	569	537, 389	232, 278	[35]
28	^B*^ Polyanoside	48.17	1071	713,489	233	[39]
29	Caffeoyl molihauside A	55.38	1137	975, 403	232, 283	[40]
31	^A*^ Ligstroside	50.46	523	477,221	228, 276	[26]
32	Caffeoyl secologanoside	59.35	551	389, 221	225,284	[38]
33	^B*^ jaspolyanoside	61.11	909	523, 233	229	[41]

A*Compounds identified from the same species

B*Compounds identified from the *Jasminum* genus

### Biological evaluation

#### MTT assay

*J. humile* extract showed a cytotoxic effect on MCF-7 breast cancer cell line with IC_50_ = 9.3 ± 1.2 µg/mL, the extract induced no cytotoxicity toward the normal cell line (HaCaT) with IC_50_ 496.2 ± 4.88 µg/mL.

#### Cell-cycle analysis

**Fig. 3:**
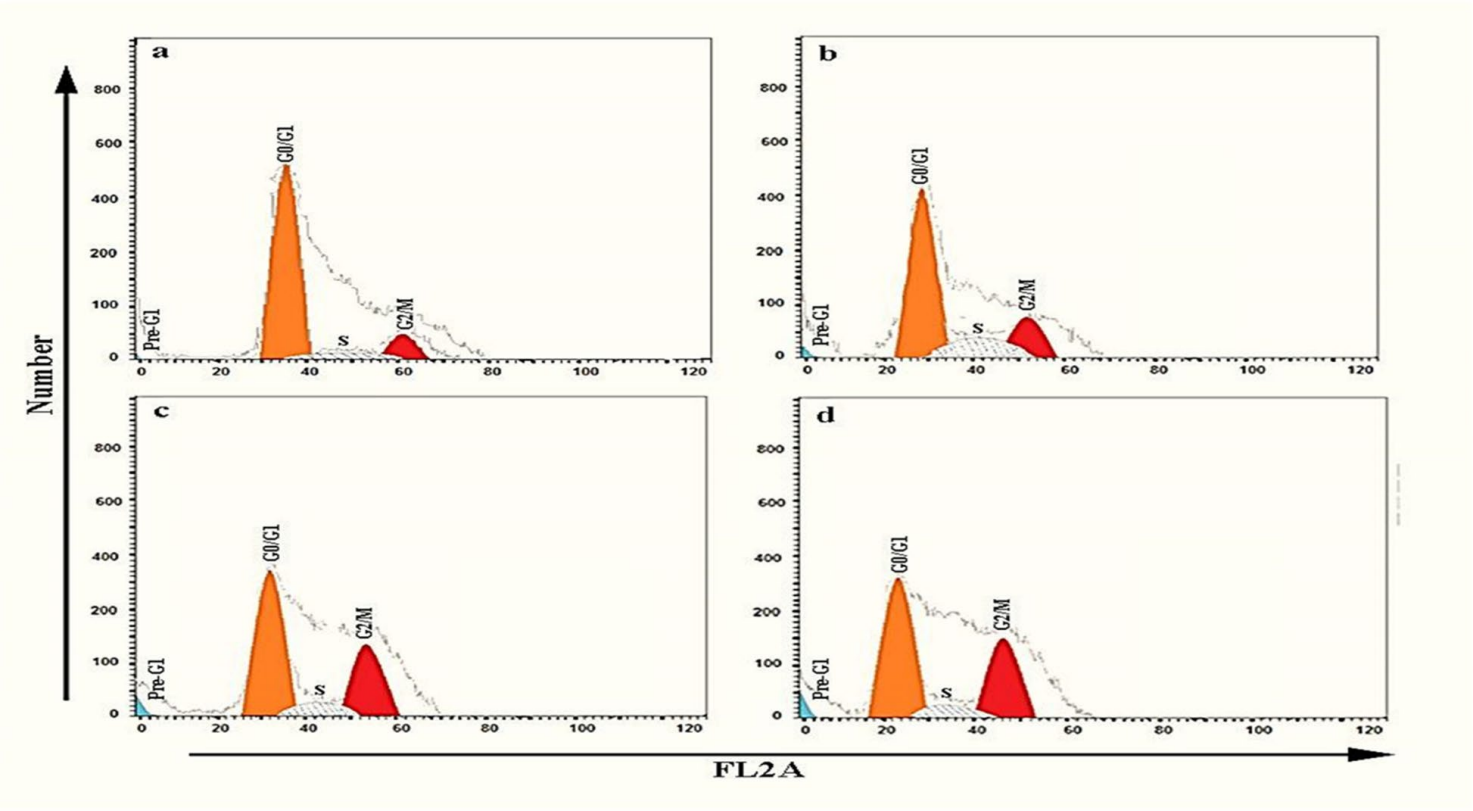
Effects of different concentrations of *J. humile* extract on Cell Cycle Analysis of MCF-7 cells. **a** Untreated MCF-7 cells, **b** MCF-7 cells treated with 5 µg/ml *J. humile* extract, **c** MCF-7 cells treated with 10 µg/ml *J. humile* extract, **d** MCF-7 cells treated with 20 µg/ml *J. humile* extract. Light blue indicates % apoptosis, orange indicates G0-G1 phase, shaded part indicates S phase and red indicates G2-M phase

**Fig. 4:**
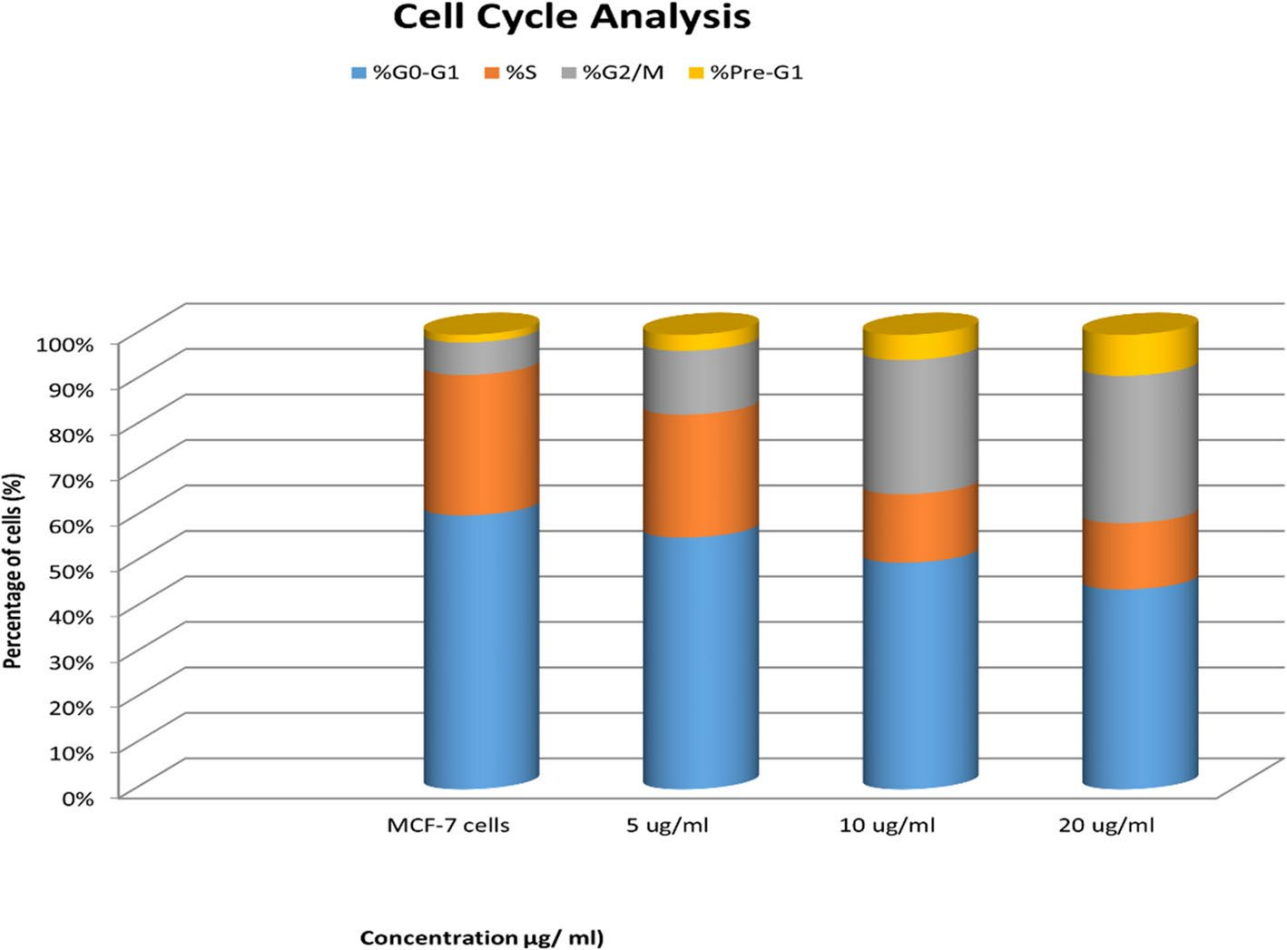
Effects of different concentrations of *J. humile* extract on Cell Cycle Analysis of MCF-7 cells

The outcome of treating MCF-7 cells with *J. humile* extract at three different concentration levels: 5, 10 and 20 µg/mL (Figs. 3 & 4) revealed that *J. humile* extract significantly reduced the number of cells in the G0-G1 and S phases compared to untreated control cells in a concentration-dependent manner, with 57.42%, 52.81%, and 48.34% compared to 61.32%, and 28.02%, 15.97% and 16.05 compared to 31.42% for 5, 10, and 20 µg/mL, respectively. Whereas data showed a significant increase in G2/M phase cell population as compared to untreated control cells in a concentration-dependent manner recording, 14.56%, 31.22%, and 35.61% as compared to 7.26% for 5, 10, and 20 µg/mL respectively. In addition, results represented a significant increase in cell cycle population in Pre-G1 phase when compared with untreated control cells in a concentration-dependent manner recording, 3.64%, 5.88%, and 9.92% compared to 1.72% for 5, 10, and 20 µg/mL, respectively.

#### Annexin V-FITC apoptosis

**Fig. 5:**
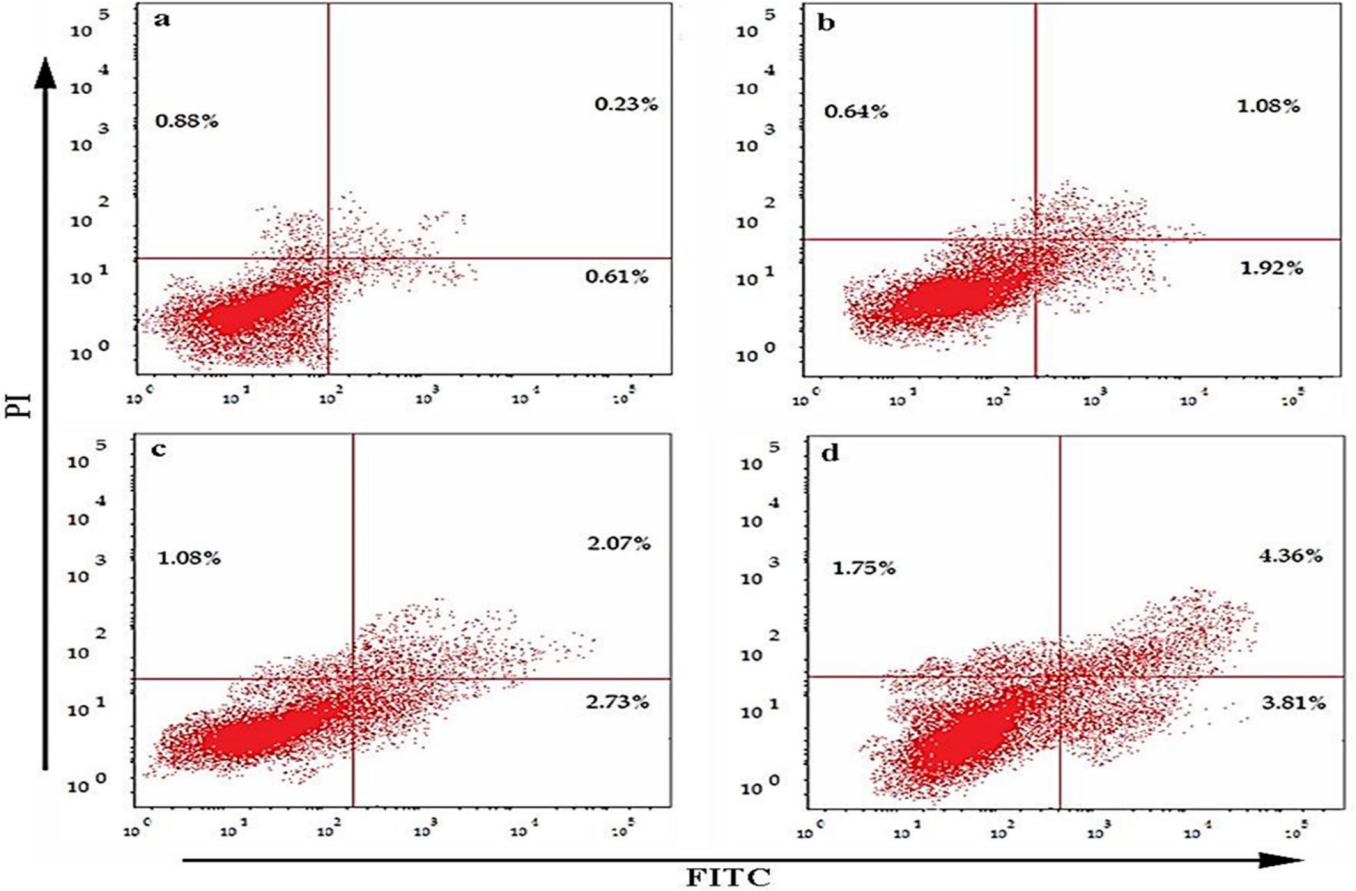
Effects of different concentrations of *J. humile* extract on Annexin V-FITC analysis of MCF-7 cells. **a** Untreated MCF-7 cells, **b** MCF-7 cells treated with 5 µg/ml *J. humile* extract, **c** MCF-7 cells treated with 10 µg/ml *J. humile* extract, **d** MCF-7 cells treated with 20 µg/ml *J. humile* extract. Early apoptosis (lower right quadrant), late apoptosis (upper right quadrant), viable cells (lower left quadrant), Necrosis (upper left quadrant). Values are presented as means ± SD with significance level at (*p* < 0.05)

**Fig. 6:**
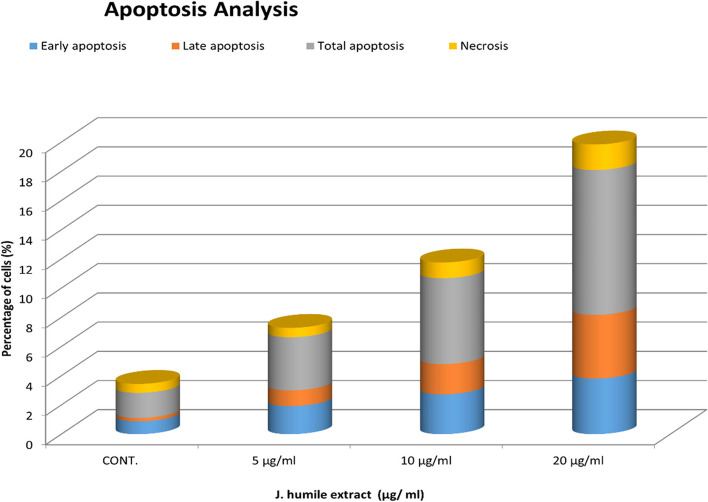
Effects of different concentrations of *J. humile* extract on rate of apoptosis and necrosis of MCF-7 cells by Annexin V-FITC

In the current study *J. humile* extract displayed cytotoxic activity against MCF-7 cancer cell line with the use of different concentrations (5, 10, and 20 µg/mL). Analyzing Fig. 3 revealed that *J. humile* extract induced a significant increase in the rate of both total apoptosis and necrosis in 3.64%, 5.88%, and 9.92% of cells at 5, 10, and 20 µg/mL, respectively compared to 1.72% in untreated control MCF-7 cells. Mutually early and late apoptosis results displayed a significant increase from control in a concentration-dependent manner (*P* < 0.05). Whereas no significant change in the rate of necrosis at 5 µg/mL (*P* < 0.05) was detected, although the 10 and 20 µg/mL showed a significant increase from control in the rate of necrosis (*P* < 0.05) as represented in (Figs. 5 & 6).

#### Oxidative stress parameters

**Fig. 7:**
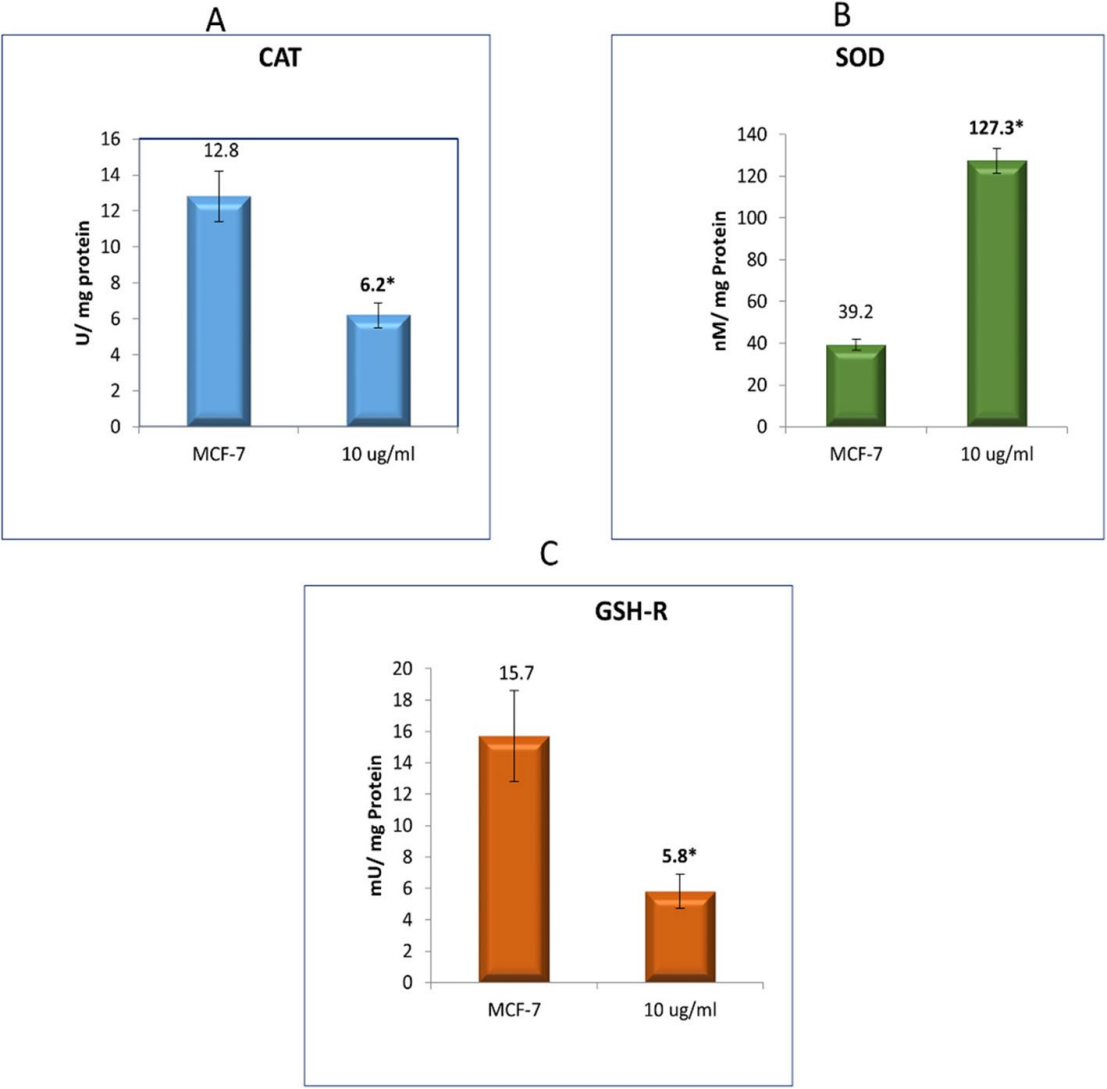
Effect of *J. humile* extract on the oxidative stress enzymes (**A**) CAT, (B) SOD and (**C**) GSH-R in MCF-7 cancer cells treated with the *J. humile* extract at 10 µg/ ml

The results in this study (Fig. 7) revealed that treatment of the MCF-7 cells with 10 µg/mL of the *J. humile* extract significantly elevated the level of SOD with concomitant depression of CAT and GSH-R as compared to the control cells causing the accumulation of ROS, thus triggering apoptosis (Table 2).

**Table 2:** Effect of *J. humile* extract on the oxidative stress enzymes CAT, SOD and GSH-R in MCF-7 cancer cells treated with the *J. humile* extract at 10 µg/ ml

Cell Groups	CAT (U/ mg protein)	SOD (nMol/ mg protein)	GSH-R (mU/ mg protein)
Untreated MCF-7 Cells	12.8 ± 1.4	39.2 ± 2.6	15.7 ± 2.9
Treated MCF-7 cells (10 µg/ ml)	6.2 ± 0.7*	127.3 ± 5.9*	5.8 ± 1.1*

Values are presented as means ± SD and (*) designates significant difference at *p* < 0.05

### Network Pharmacology

#### Scrutinizing anti-breast cancer target

**Fig. 8:**
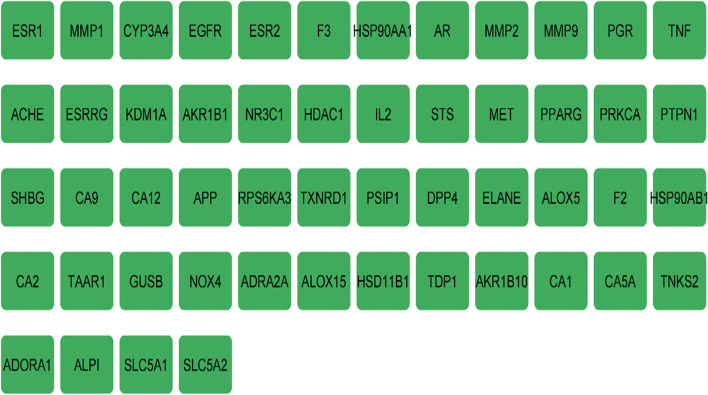
Names of overlapping genes

**Fig. 9:**
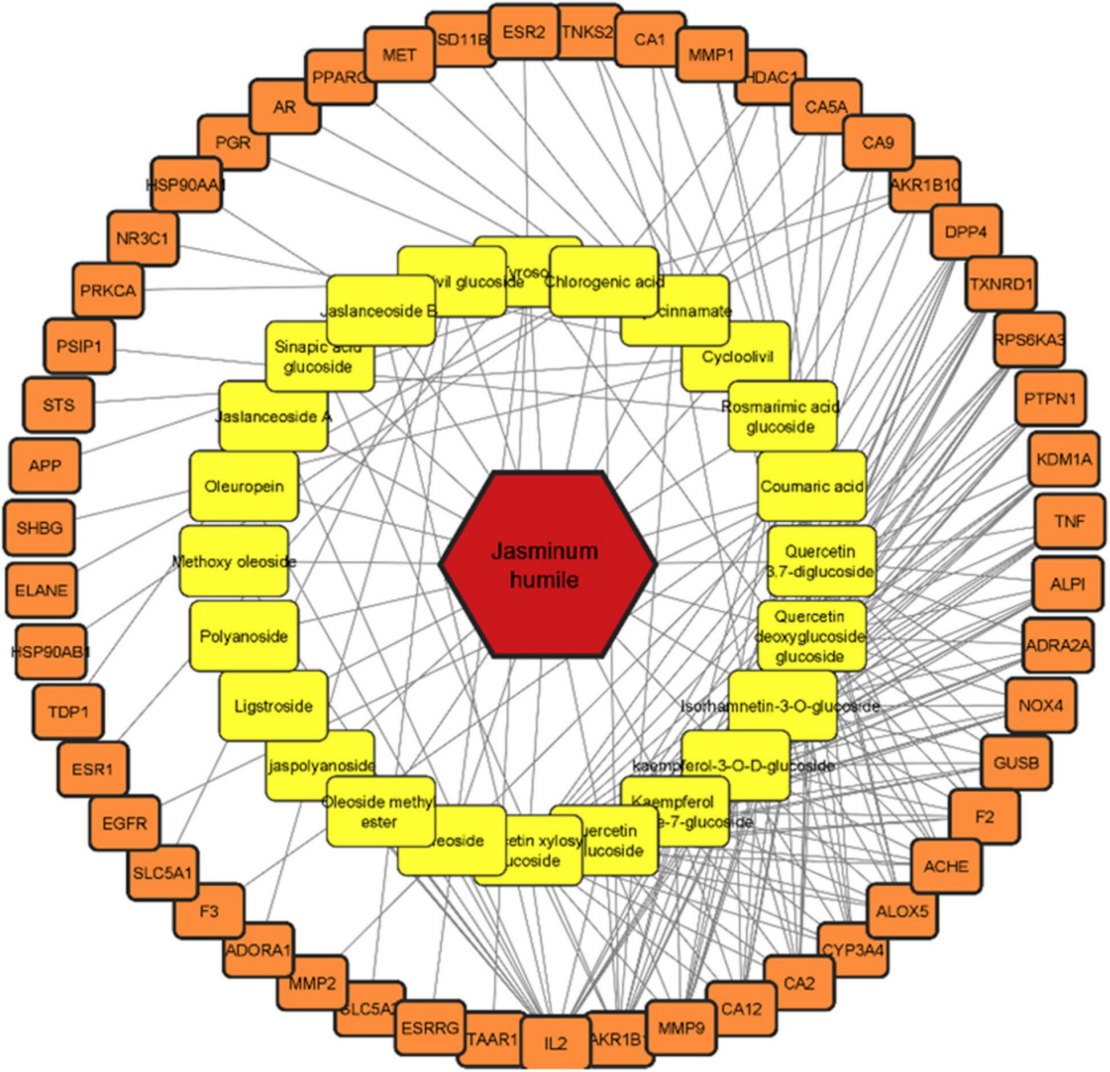
Network diagram of active compounds-target gene

A total of 231 genes were related to 24 compounds from the above-mentioned 33 compounds using Binding DB prediction (Table S1), while 9 compounds exhibited no human target interactions. A total of 6776 disease-related genes were found after retrieving the DisGeNET database. 52 overlapped genes were discovered after comparing 231 compound-related genes with 6776 disease-related genes. The names of overlapping genes targeted by *J.humile* drugs and breast cancer are shown in Fig. 8. A network was constructed for active compounds and overlapping genes which includes 76 nodes and 225 edges (Fig. 9).

#### GO and KEGG analysis

**Fig. 10:**
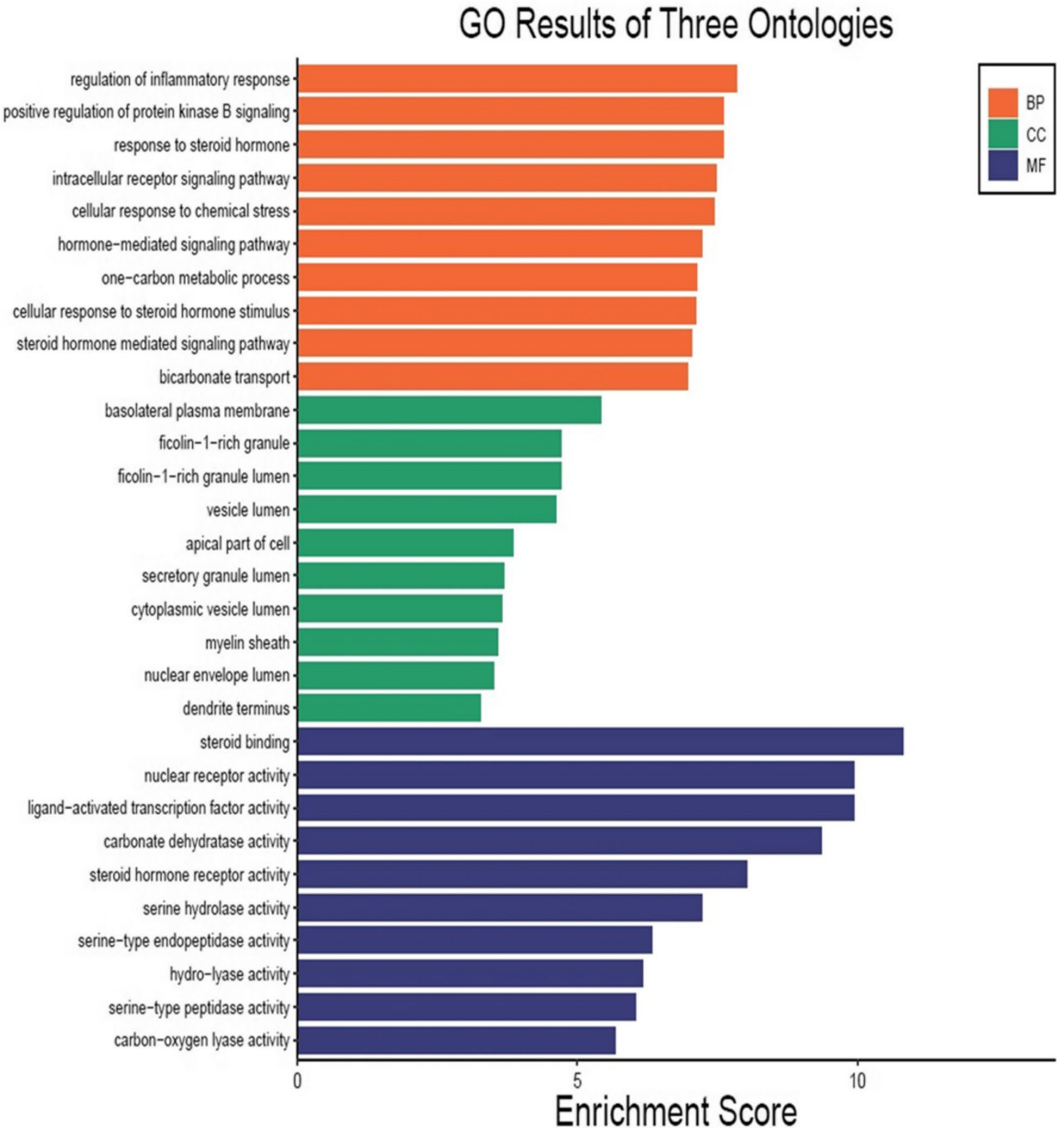
Top 10 GO enrichment pathways. X-axis is enrichment gene ratio, Y-axis is biological process (BP), Cellular components (CC) and Molecular function (MF)

**Fig. 11:**
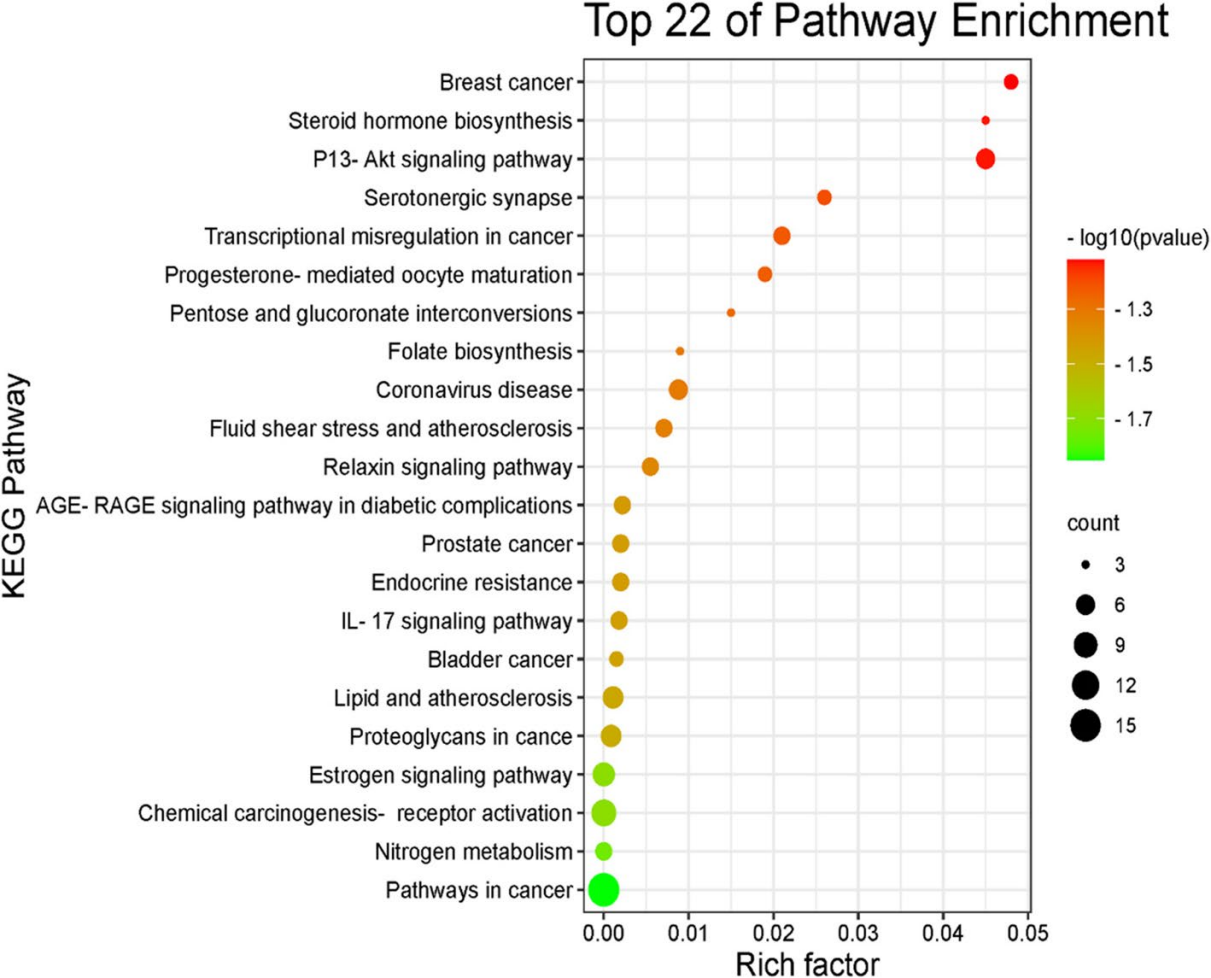
KEGG pathway. X-axis is enrichment gene count, Y-axis is KEGG pathway, and the color of bar chart represents the adjusted *p*-value

**Fig. 12:**
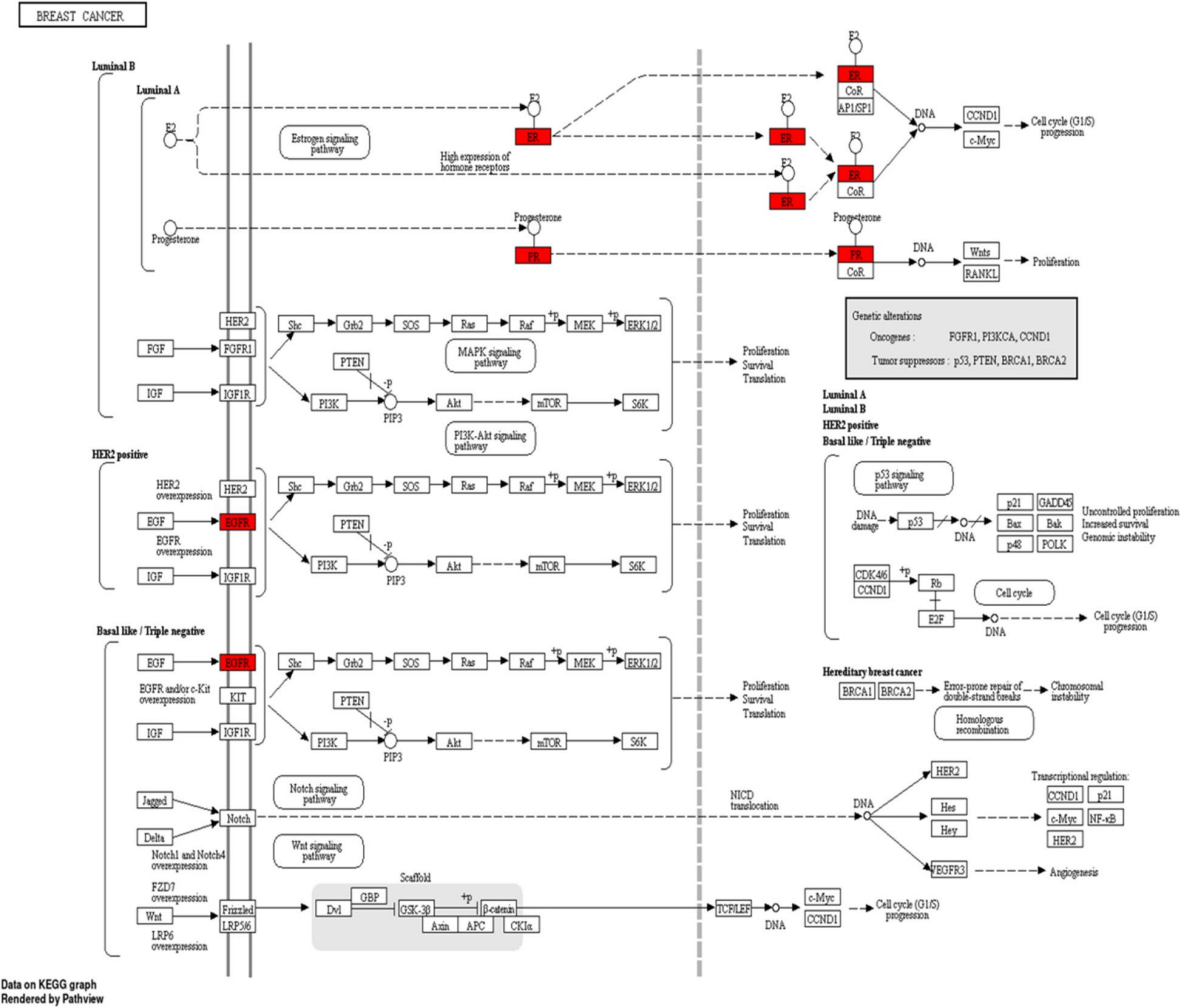
KEGG pathway for breast cancer (hsa05224), figure obtained under permission and guidelines [42]

To show the interaction of target proteins with their relevant components, DAVID analysis of GO enrichment and KEGG analysis were used. The Benjamini–Hochberg procedure was used to correct *P*-values, and the top 10 significantly enriched items in the BP, MF, and CC categories (Fig. 10 & Table S2) were picked based on p ≤ 0.05. According to GO functional analysis, *J. humile's* principal targets were inflammatory response regulation, protein kinase B regulation, steroid hormone response, intracellular receptor signaling pathway, and so on. Relevant signaling pathways related with *J. humile's* anti-breast cancer action were identified using KEGG pathway analysis. Pathways in cancer (16), chemical carcinogenesis-receptor activation (10), estrogen signaling system (8), proteoglycans in cancer (7), lipid and atherosclerosis (6), and P13-Akt signaling pathway (6) had the most genes (Table S3). The substantially enriched genes from the 22 KEGG signaling pathways results (Fig. 11) were EGFR, ESR1, ESR2, HSP90AA1, HSP90AB1, MMP9, and MMP1 [42]. Breast cancer pathways had been targeted by target genes thereby KEGG pathway for breast cancer is depicted in Fig. 12, where target genes were represented with red color. On the basis of this pathway, it can be concluded that *J. humile* exerts the anti-breast cancer effect by targeting the estrogen signaling pathway, HER2 proteins, and triple-negative breast cancer process.

### Molecular docking

For molecular docking, the top most hub gene EGFR was selected based on PPI interactions analysis. The top most active compounds Quercetin 3,7-diglucoside, Quercetin xylosyl glucoside, Kaempferol 3-xyloside-7-glucoside, kaempferol-3-O-D-glucoside and Isorhamnetin-3-O-glucoside were docked with EGFR. The two crucial factors were used while analyzing docking results: (i) the best docked pose binding energy prediction using MOE scoring system and (ii) Hydrogen bond information of top-ranked posture. Each compound was docked in 10 distinct positions throughout the docking run. The recovered molecules were first sorted using the pre-validated methodology described above, and then the visualization approach was used to determine the inhibitor binding mode that is best based on the inhibitor's critical interactions with the active site residues. Table 3 summarizes the docking information for the top-ranked poses.

**Table 3:** Putative Binding mode interaction of Top most compounds with EGFR

Compound Name	Binding Energy (KJ/mol)	Bond length (Å)	Amino acids
Quercetin3,7-diglucoside	-14.6360	2.91	Thr766
		2.88	Thr766
		2.69	Thr830
		2.52	Lys721
Quercetinxylosyl glucoside	-14.6385	2.45	Glu738
		2.63	Glu738
		2.77	Lys721
		1.84	Lys721
Kaempferol 3-xyloside-7-glucoside	-15.1710	2.68	Lys692
		2.58	Lys692
		1.76	Lys692
		2.5	Asp831
		2.54	Thr830
kaempferol-3-O-D-glucoside	-17.7126	2.79	Thr766
		3.14	Thr830
		3.24	Lys721
Isorhamnetin-3-O-glucoside	-16.2629	2.66	Glu738
		2.38	Thr766
		2.73	Asp831
		2.29	Lys721
		2.81	Lys721
		2.62	Thr766
		2.38	Thr766
		2.44	Thr830

**Fig. 13:**
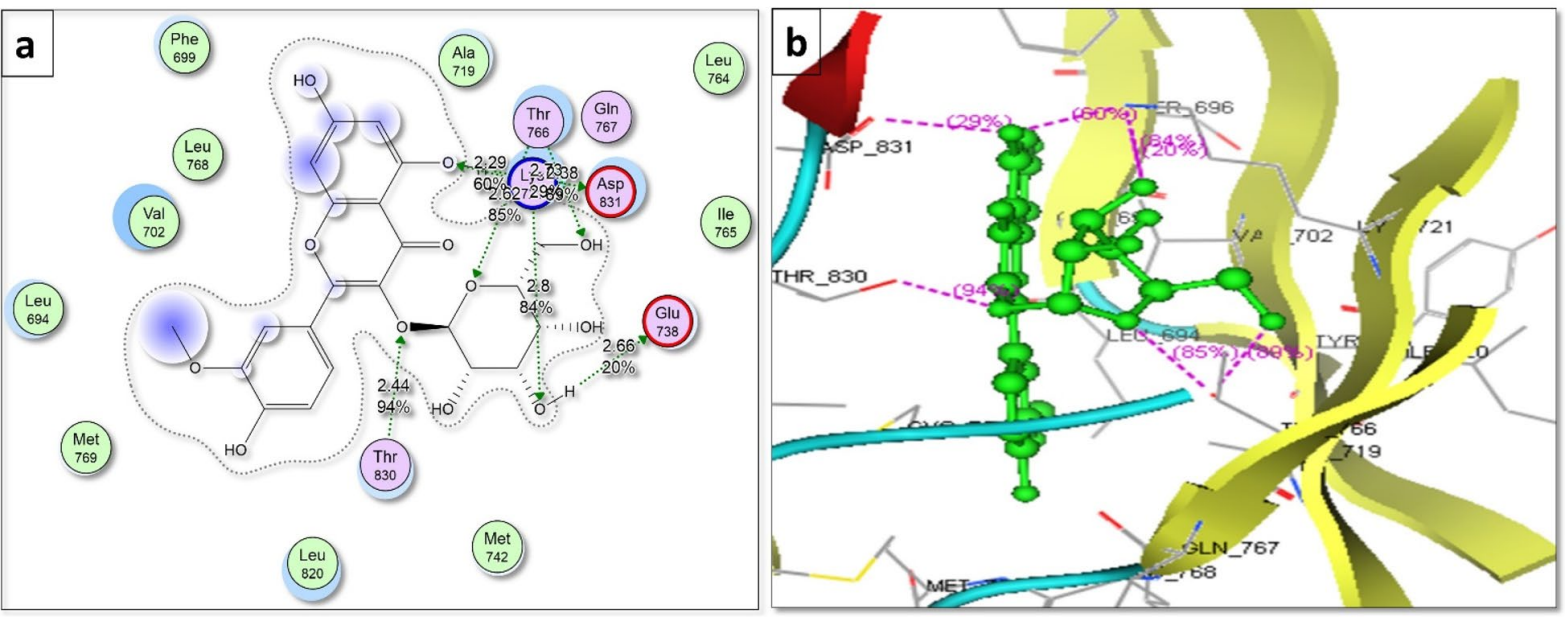
Binding mode of Isorhamnetin-3-O-glucoside with EGFR; **a**: 2D and **b**: 3D

All the selected compounds displayed significant interaction with the EGFR. All of them bind in the same binding pocket where the attached inhibitor was present thus showing that these compounds also possess inhibitory attributes on EGFR. Compounds interacted with the active site of EGFR by forming bonds with the following amino acid residues: Thr766, Thr830, Lys 721, Glu738, Lys 692, Asp 831 and Thr 830. Among all compounds, Isorhamnetin-3-O-glucoside displayed maximum binding interaction with EGFR as shown in Fig. 13.

## Discussion

Breast cancer is considered one of the most common causes of cancer related death among women [43]. Extracts of several species of the genus *Jasminum* revealed different pharmacological activities as anticancer [9–11]. In the present study, we evaluated the effect of *Jasminum humile* on breast cancer cells. Our results demonstrated that *J. humile* significantly inhibited breast cancer growth and was inactive on healthy cells suggesting a potential selective anti-cancer activity of the extract. *J. humile* extract caused a dose-dependent inhibition of cell growth with S phase arrest, a reduction in G0/G1 phase, and an increase in pre-G1 cells. An increase of cells in the pre-G1 phase was recognized as an indicator of DNA fragmentation and hence apoptosis. The results could prove that *J. humile* extract may exert its cytotoxic effect in MCF-7 cells by arresting its proliferation in G2/M phase. In order to determine if cell death occurred through apoptosis or necrosis Annexin V-based flow cytometry analysis was performed. Annexin-V FTIC assay showed that *J. humile* extract displayed a noticeable rise in apoptotic cell percentage including both early and late apoptotic phases in a concentration-dependent manner. Additionally, humile extract had a cytotoxic effect by interfering with the redox homeostasis of the cell between ROS production and the antioxidant system. All these results indicate that *J. humile* may serve as a potential therapeutic agent against breast cancer, however the identities of the effective compounds remain unclear. HPLC–PDA-MS/MS metabolites profiling identified 33 compounds. Utilizing network pharmacology, out of 33 compounds, 24 displayed interaction with 52 human target genes as potential target of *J. humile* responsible for its inhibitory effect against breast cancer. Then, DAVID analysis of GO enrichment and KEGG analysis were used to show the interaction of target proteins with their relevant components. According to GO functional analysis, *J. humile's* principal targets were inflammatory response regulation, protein kinase B regulation, steroid hormone response, intracellular receptor signaling pathway, and so on. Relevant signaling pathways related with *J. humile's* anti-breast cancer action were identified using KEGG pathway analysis. Pathways in cancer (16), chemical carcinogenesis-receptor activation (10), estrogen signaling system (8), proteoglycans in cancer (7), lipid and atherosclerosis (6), and P13-Akt signaling pathway (6) had the most genes. The substantially enriched genes from the 22 KEGG signaling pathways results were EGFR, ESR1, ESR2, HSP90AA1, HSP90AB1, MMP9, and MMP1. On the basis of this pathway, it can be concluded that *J. humile* exerts the anti-breast cancer effect by targeting the estrogen signaling pathway, HER2 proteins, and triple-negative breast cancer process. Notably, the epidermal growth factor receptor (EGFR) signaling pathway is one of the most important pathways that regulate growth, survival, proliferation, and differentiation in mammalian cells [44]. EGFR is a receptor tyrosine kinase that is commonly upregulated in cancers such as in non-small-cell lung cancer, metastatic colorectal cancer, glioblastoma, head and neck cancer, pancreatic cancer, and breast cancer [45]. To further verify the results of network pharmacology, molecular docking was performed with the five key compounds (Quercetin 3,7-diglucoside, Quercetin xylosyl glucoside, Kaempferol 3-xyloside-7-glucoside, kaempferol-3-O-D-glucoside and Isorhamnetin-3-O-glucoside) and the top most target, EGFR. All the selected compounds displayed significant interaction with the EGFR. Among all compounds, Isorhamnetin-3-O-glucoside displayed maximum binding interaction with EGFR. Interestingly, isorhamnetin-3-O-rhamnoside showed strong inhibitory effects on human breast adenocarcinoma cell line MCF-7 proliferation [46]. The results of molecular docking were consistent with those of network pharmacology.

## Conclusions

In conclusion, our study provides the first clear evidence that *Jasminum humile* L. flowers have significant antitumor activity against breast cancer. Furthermore, *Jasminum humile* L. significantly inhibited cell growth, prompted cell apoptosis, and caused cell cycle arrest partly via EGFR signaling pathway. Additionally, we identified many active compounds from *Jasminum humile* that can be used to develop new therapeutic agents to fight breast cancer.

## Supplementary Information

**Additional file 1:** **Table S1.** Genes related to 24compounds. **Table S2.** GO Analysis. **Table S3.** KEGG Pathwaysanalysis.

## Data Availability

All data generated or analysed during this study are included in this published article [and its supplementary information files].
