# Correction: Network pharmacology and molecular docking study for biological pathway detection of cytotoxicity of the yellow jasmine flowers

**DOI:** 10.1186/s12906-023-04011-x

**Published:** 2023-05-31

**Authors:** Seham S. El‑Hawary, Marzough A. Albalawi, Ayat O. S. Montasser, Shaimaa R. Ahmed, Sumera Qasim, Ali A. Shati, Mohammad Y. Alfaifi, Serag Eldin I. Elbehairi, Omnia F. Hassan, Abdelfattah A. Sadakah, Fatma A. Mokhtar

**Affiliations:** 1 Department of Pharmacognosy, Faculty of Pharmacy, Cairo University, Kasr El‑Aini Street, Cairo, Egypt; 2 Department of Chemistry, Alwajh College, University of Tabuk, Tabuk, 71491 Saudi Arabia; 3 National Organization for Drug Control (NODCAR), Cairo, Egypt; 4 Department of Pharmacognosy, College of Pharmacy, Jouf University, Sakaka, 72341 Aljouf Saudi Arabia; 5 Department of Pharmacology, College of Pharmacy, Jouf University, Sakaka, 72341 Aljouf Saudi Arabia; 6 King Khalid University, Faculty of Science, Biology Department, Abha, 9004 Saudi Arabia; 7 Department of Pharmacology and Toxicology, Faculty of Pharmacy, MSA University, 6th of October City, Egypt; 8 Oral and Maxillofacial Surgery Department, Faculty of Dentistry, Tanta University, Tanta, Egypt; 9 Oral and Maxillofacial Surgery Department, Faculty of Dentistry, ALsalam University, Kafr Alzayat, Al Gharbia, Egypt; 10 Department of Pharmacognosy, Faculty of Pharmacy, Al Salam University, Kafr Alzayat, Al Gharbia, Egypt; 11 Department of Pharmacognosy, Faculty of Pharmacy, El Saleheya El Gadida University, Sharkia 44813 El Saleheya El Gadida, Egypt

**Correction: BMC Complement Med Ther 23, 164 (2023)**

**https://doi.org/10.1186/s12906-023-03987-w**

Following publication of the original article [1], the authors would like to remove the affiliation ‘Cell Culture Lab, Egyptian Organization for Biological Products and Vaccines (VACSERA Holding Company), Giza, Egypt*’* to author Serag Eldin I. Elbehairi.

The author group has been updated above and the original article has been corrected.
