# Supplementary material for: Network pharmacology and molecular docking study for biological pathway detection of cytotoxicity of the yellow jasmine flowers
Source: BMC Complement Med Ther. 2023 May 20;23:164. doi: 10.1186/s12906-023-03987-w (PMC10199617; doi:10.1186/s12906-023-03987-w)
Supplement: Supplementary file 1 — Additional file 1: Table S1. Genes related to 24compounds. Table S2. GO Analysis. Table S3. KEGG Pathwaysanalysis. [file 12906_2023_3987_MOESM1_ESM.docx]

**Supplementary Data**

**Network Pharmacology and Molecular docking study for biological pathway detection of cytotoxicity of the yellow jasmine flowers**

Seham S. El-Hawary^1^, Marzough A albalawi^2^, Ayat OS. Montasser^3^, Shaimaa R. Ahmed^4^, Sumera Qasim^5^, Ali A. Shati^6^, Mohammad Y. Alfaifi^6^, Serag Eldin I. Elbehairi^6,7^, Omnia F. Hassan^8^ , Abdelfattah A. Sadakah^9,10^ , Fatma A. Mokhtar^11,12🖂^

^1^Department of Pharmacognosy, Faculty of Pharmacy, Cairo University, Kasr El-Aini Street, Cairo, Egypt.

^2^Department of Chemistry, Alwajh college, University of Tabuk, Tabuk 71491, Saudi Arabia

^3^National organization for drug control (NODCAR), Cairo, Egypt.

^4^Phytochemistry and Plant Systematic Department, National Research Centre, Dokki, Cairo, Egypt.

^5^ Department of Pharmacognosy, College of Pharmacy, Jouf University, Sakaka, Aljouf 72341, Saudi Arabia

^5^ Department of Pharmacology, College of Pharmacy, Jouf University, Sakaka, Aljouf 72341, Saudi Arabia

^6^ King Khalid University, Faculty of Science, Biology Department, Abha 9004, Saudi Arabia

^7^ Cell Culture Lab, Egyptian Organization for Biological Products and Vaccines (VACSERA Holding Company), Giza, Egypt

^8^ Department of Pharmacology and Toxicology, Faculty of Pharmacy, MSA University, 6th of October City, Egypt.

^9^ Oral and Maxillofacial Surgery Department, Faculty of Dentistry, Tanta University, Tanta, Egypt

^10^ Oral and Maxillofacial Surgery Department, Faculty of Dentistry, ALsalam University, Kafr Alzayat, Al Gharbia, Egypt.

^11^Department of Pharmacognosy, Faculty of Pharmacy, Al Salam University, kafr alzayat, Al Gharbia, Egypt

^12^Department of pharmacognosy, Faculty of pharmacy, El Saleheya El Gadida University, El Saleheya El Gadida 44813, Sharkia, Egypt.

^🖂^ Corresponding author:

Fatma A. Mokhtar Ali

email: [Fatma.Mokhtar@sue.edu.eg](mailto:Fatma.Mokhtar@sue.edu.eg)

Orcid: https://orcid.org/0000-0002-6909-7440

**Abstract**

**Background:** The yellow jasmine flower (*Jasminum humile* L.) is a fragrant plant belonging to the Oleaceae family with promising phytoconstituents and interesting medicinal uses. The purpose of this study was to characterize the plant metabolome to identify the potential bioactive agents with cytotoxic effects and the underlying mechanism of cytotoxic activity.

**Methods:** First, HPLC-PDA-MS/MS was used to identify the potential bioactive compounds in the flowers. Furthermore, we assessed the cytotoxic activity of the flower extract against breast cancer (MCF-7) cell line using MTT assay followed by the cell cycle, DNA-flow cytometry, and Annexin V-FITC analyses alongside the effect on reactive oxygen species (ROS). Finally, Network pharmacology followed by a molecular docking study was performed to predict the pathways involved in anti-breast cancer activity.

**Results:** HPLC-PDA-MS/MS tentatively identified 33 compounds, mainly secoiridoids. *J. humile* extract showed a cytotoxic effect on MCF-7 breast cancer cell line with IC_50_ value of 9.3 ±1.2 µg/mL. Studying the apoptotic effect of J. humile extract revealed that it disrupts G2/M phase in the cell cycle, increases the percentage of early and late apoptosis in Annexin V-FTIC, and affects the oxidative stress markers (CAT, SOD, and GSH-R). Network analysis revealed that out of 33 compounds, 24 displayed interaction with 52 human target genes. Relationship between compounds, target genes, and pathways revealed that *J. humile* exerts its effect on breast cancer by altering, Estrogen signaling pathway, HER2, and EGFR overexpression. To further verify the results of network pharmacology, molecular docking was performed with the five key compounds and the topmost target, EGFR. The results of molecular docking were consistent with those of network pharmacology.

**Conclusion:** Our findings suggest that *J. humile* suppresses breast cancer proliferation and induces cell cycle arrest and apoptosis partly by EGFR) signaling pathway, highlighting *J. humile* as a potential therapeutic candidate against breast cancer.

**Keywords:** Apoptosis; LC/MS/MS; *Jasminum humile;* MCF-7; Oleaceae; network pharmacology, molecular docking.

**Table S1: Genes related to 24 compounds**

| Tyrosol | ESR1 | ESR2 | ACHE | ESRRG | TAAR1 |  |  |  |  |  |  |  |  |  |  |  |  |  |  |
| --- | --- | --- | --- | --- | --- | --- | --- | --- | --- | --- | --- | --- | --- | --- | --- | --- | --- | --- | --- |
| Rosmarimic acid glucoside | MMP1 | MMP9 | AKR1B1 | MET | PPARG | CA9 | CA12 | PSIP1 | ALOX5 | AKR1B10 | CA5A |  |  |  |  |  |  |  |  |
| Coumaric acid | MMP1 | EGFR | F3 | MMP2 | MMP9 | AKR1B1 | HDAC1 | CA9 | CA12 | CA2 | HSD11B1 | AKR1B10 | CA1 | CA5A | TNKS2 |  |  |  |  |
| Cycloolivil | CYP3A4 | ESR2 | AR | PGR | ACHE | NR3C1 | STS | SHBG |  |  |  |  |  |  |  |  |  |  |  |
| Quercetin 3,7-diglucoside | CYP3A4 | MMP9 | TNF | KDM1A | AKR1B1 | IL2 | PTPN1 | CA12 | RPS6KA3 | TXNRD1 | DPP4 | ALOX5 | F2 | CA2 | GUSB | NOX4 | ADRA2A | ALPI |  |
| Quercetin xylosyl glucoside | CYP3A4 | MMP9 | TNF | ACHE | KDM1A | AKR1B1 | IL2 | PTPN1 | CA12 | RPS6KA3 | TXNRD1 | DPP4 | ALOX5 | F2 | CA2 | GUSB | NOX4 | ADRA2A | ALPI |
| Quercetin 3-glucoside | CYP3A4 | MMP9 | TNF | ACHE | KDM1A | AKR1B1 | IL2 | PTPN1 | CA12 | RPS6KA3 | TXNRD1 | DPP4 | ALOX5 | F2 | CA2 | GUSB | NOX4 | ADRA2A | ALPI |
| Kaempferol 3-xyloside-7-glucoside | CYP3A4 | MMP9 | TNF | ACHE | KDM1A | AKR1B1 | IL2 | PTPN1 | CA12 | RPS6KA3 | TXNRD1 | DPP4 | ALOX5 | F2 | CA2 | GUSB | NOX4 | ADRA2A | ALPI |
| kaempferol-3-O-D-glucoside | CYP3A4 | MMP9 | TNF | ACHE | KDM1A | AKR1B1 | IL2 | PTPN1 | CA12 | RPS6KA3 | TXNRD1 | DPP4 | ALOX5 | F2 | CA2 | GUSB | NOX4 | ADRA2A | ALPI |
| Isorhamnetin-3-O-glucoside | CYP3A4 | MMP9 | TNF | ACHE | KDM1A | AKR1B1 | IL2 | PTPN1 | CA12 | RPS6KA3 | TXNRD1 | DPP4 | ALOX5 | F2 | CA2 | GUSB | NOX4 | ADRA2A | ALPI |
| Quercetin deoxyglucoside glucoside | CYP3A4 | MMP9 | TNF | CHE | KDM1A | AKR1B1 | IL2 | PTPN1 | CA12 | RPS6KA3 | TXNRD1 | DPP4 | ALOX5 | F2 | CA2 | GUSB | NOX4 | ADRA2A | ALPI |
| Jaslanceoside B | HSP90AA1 | IL2 |  |  |  |  |  |  |  |  |  |  |  |  |  |  |  |  |  |
| Chlorogenic acid | AKR1B1 | HDAC1 | PRKCA | APP | ELANE | HSP90AB1 | AKR1B10 |  |  |  |  |  |  |  |  |  |  |  |  |
| Ethyl cinnamate | AKR1B1 | HDAC1 | CA9 | CA2 | AKR1B10 | CA1 | CA5A | TNKS2 |  |  |  |  |  |  |  |  |  |  |  |
| Sinapic acid glucoside | AKR1B1 | TDP1 |  |  |  |  |  |  |  |  |  |  |  |  |  |  |  |  |  |
| Oleoside methyl ester | IL2 |  |  |  |  |  |  |  |  |  |  |  |  |  |  |  |  |  |  |
| Oleoside | IL2 |  |  |  |  |  |  |  |  |  |  |  |  |  |  |  |  |  |  |
| Jaslanceoside A | IL2 |  |  |  |  |  |  |  |  |  |  |  |  |  |  |  |  |  |  |
| Oleuropein | IL2 |  |  |  |  |  |  |  |  |  |  |  |  |  |  |  |  |  |  |
| Methoxy oleoside | IL2 |  |  |  |  |  |  |  |  |  |  |  |  |  |  |  |  |  |  |
| Polyanoside | IL2 |  |  |  |  |  |  |  |  |  |  |  |  |  |  |  |  |  |  |
| Ligstroside | IL2 |  |  |  |  |  |  |  |  |  |  |  |  |  |  |  |  |  |  |
| jaspolyanoside | IL2 |  |  |  |  |  |  |  |  |  |  |  |  |  |  |  |  |  |  |
| Cycloolivil glucoside | SLC5A1 | ADORA1 | SLC5A2 |  |  |  |  |  |  |  |  |  |  |  |  |  |  |  |  |

**Table S2: GO Analysis**

**(I) Top 10 cellular components linked to target genes**

| **Description** | **pvalue** | **geneID** | **Count** |
| --- | --- | --- | --- |
| regulation of inflammatory response | 1.12982E-08 | ESR1/ADORA1/EGFR/ALOX15/IL2/ELANE/MMP9/PPARG/ALOX5/F2/TNF | 11 |
| response to steroid hormone | 2.02517E-08 | ESR1/CA2/ESRRG/EGFR/ESR2/NR3C1/HDAC1/AR/PGR/TNF | 10 |
| positive regulation of protein kinase B signaling | 2.05382E-08 | ESR1/EGFR/NOX4/F3/HSP90AA1/MET/TNF/HSP90AB1 | 8 |
| intracellular receptor signaling pathway | 2.81592E-08 | ESR1/ESRRG/ESR2/NR3C1/HDAC1/ALOX15/AR/PPARG/PGR | 9 |
| cellular response to chemical stress | 2.94509E-08 | EGFR/AKR1B1/NOX4/TXNRD1/MMP2/MET/MMP9/PPARG/ALOX5/TNF | 10 |
| hormone-mediated signaling pathway | 5.08339E-08 | ESR1/ESRRG/ESR2/NR3C1/HDAC1/AR/PPARG/PGR | 8 |
| cellular response to steroid hormone stimulus | 6.38971E-08 | ESR1/ESRRG/EGFR/ESR2/NR3C1/HDAC1/AR/PGR | 8 |
| steroid hormone mediated signaling pathway | 7.76372E-08 | ESR1/ESRRG/ESR2/NR3C1/HDAC1/AR/PGR | 7 |
| Bicarbonate transport | 1.03784E-07 | ADORA1/EGFR/APP/ADRA2A/HSP90AA1/MMP9/PRKCA | 7 |
| One carbon metabolic process | 2.77547E-07 | EGFR/APP/NOX4/TXNRD1/PSIP1/MMP2/MET/MMP9/ALOX5/TNF | 10 |

**(II) Top 10 biological process linked to target genes**

| **Description** | **pvalue** | **geneID** | **Count** |
| --- | --- | --- | --- |
| basolateral plasma membrane | 3.30287E-06 | CA2/ADORA1/CA9/EGFR/ADRA2A/MET/HSP90AB1 | 7 |
| ficolin-1-rich granule | 1.75672E-05 | GUSB/HSP90AA1/MMP9/ALOX5/HSP90AB1 | 5 |
| ficolin-1-rich granule lumen | 1.75672E-05 | GUSB/HSP90AA1/MMP9/ALOX5/HSP90AB1 | 5 |
| vesicle lumen | 2.15005E-05 | GUSB/EGFR/APP/HSP90AA1/ELANE/ALOX5/HSP90AB1 | 7 |
| apical part of cell | 0.000123968 | CA2/SLC5A1/EGFR/APP/NOX4/DPP4/HSP90AB1 | 7 |
| secretory granule lumen | 0.000183442 | GUSB/APP/HSP90AA1/ELANE/ALOX5/HSP90AB1 | 6 |
| cytoplasmic vesicle lumen | 0.000196105 | GUSB/APP/HSP90AA1/ELANE/ALOX5/HSP90AB1 | 6 |
| myelin sheath | 0.000249766 | CA2/AKR1B1/HSP90AA1 | 3 |
| nuclear envelope lumen | 0.00029599 | APP/ALOX5 | 2 |
| dendrite terminus | 0.000510487 | HSP90AA1/HSP90AB1 | 2 |

**(III) Top 10 Molecular functions linked to target genes**

| **Description** | **pvalue** | **geneID** | **Count** |
| --- | --- | --- | --- |
| steroid binding | 1.25902E-11 | ESR1/SHBG/ESRRG/CYP3A4/ESR2/NR3C1/HSD11B1/AR/PGR | 9 |
| nuclear receptor activity | 1.01413E-10 | ESR1/ESRRG/ESR2/NR3C1/AR/PPARG/PGR | 7 |
| ligand-activated transcription factor activity | 1.01413E-10 | ESR1/ESRRG/ESR2/NR3C1/AR/PPARG/PGR | 7 |
| steroid hormone receptor activity | 8.52936E-09 | ESR1/ESRRG/ESR2/NR3C1/PGR | 5 |
| serine hydrolase activity | 5.16198E-08 | ACHE/MMP1/F3/DPP4/MMP2/ELANE/MMP9/F2 | 8 |
| carbonate dehydratase activity | 7.05863E-08 | CA2/CA9/CA12/CA5A | 4 |
| serine-type endopeptidase activity | 4.08213E-07 | MMP1/F3/DPP4/MMP2/ELANE/MMP9/F2 | 7 |
| serine-type peptidase activity | 8.08044E-07 | MMP1/F3/DPP4/MMP2/ELANE/MMP9/F2 | 7 |
| hydro-lyase activity | 2.15066E-05 | CA2/CA9/CA12/CA5A | 4 |
| carbon-oxygen lyase activity | 5.27286E-05 | CA2/CA9/CA12/CA5A | 4 |

**Table S3: KEGG Pathways analysis**

| **KEGG pathway** | **Enrichment** | **P value** | **Gene count** | **Genes** |
| --- | --- | --- | --- | --- |
| Pathways in cancer | 0.000000079 | 71.02 | 16 | MET,AR, F2,EGFR,ESR1, ESR2,HSP90AA1,HSP90AB1,HDAC1,  IL2,MMP1,MMP2,MMP9,PPARG,PRKCA,TXNRD1 |
| Nitrogen metabolism | 0.0000019 | 57.21 | 5 | CA12. CA2, CA5A, CA9 |
| Chemical carcinogenesis- receptor activation | 0.0000094 | 50.26 | 10 | AR,EGFR,ESR1,ESR2,HSP90AA1,HSP90AB1,PGR,PRKCA,RPS6KA3 |
| Estrogen signaling pathway | 0.0000094 | 50.26 | 8 | EGFR,ESR1,ESR2,HSP90AA1,HSP90AB1,MMP2,MMP,PGR |
| Proteoglycans in cancer | 0.00087 | 30.6 | 7 | MET,EGFR,ESR1,MMP2,MMP9,PRKCA,TNF |
| Lipid and atherosclerosis | 0.0011 | 29.58 | 7 | HSP90AA1,HSP90AB1,MMP1,MMP9,PPARG,PRKCA,TNF |
| Bladder cancer | 0.0015 | 28.23 | 4 | EGFR,MMP1,MMP2,MMP9 |
| IL-17 signaling pathway | 0.0018 | 27.44 | 5 | HSP90AB1,HSP90AA1,MMP1,MMP9.TNF |
| Prostate cancer | 0.002 | 26.98 | 5 | AR,EGFR, HSP90AB1,HSP90AA1,MMP |
| Endocrine resistance | 0.002 | 26.98 | 5 | EGFR,ESR2, ESR1,MMP2,MMP9 |
| AGE-RAGE signaling pathway in diabetic complications | 0.0022 | 26.57 | 5 | NOX4,F3,MMP2,PRKCA,TNF |
| Relaxin signaling pathway | 0.0055 | 22.59 | 5 | \| EGFR \| MMP1 \| MMP2 \| MMP9 \| PRKCA \| \| --- \| --- \| --- \| --- \| --- \| |
| Fluid shear stress and atherosclerosis | 0.0071 | 21.48 | 5 | \| HSP90AB1 \| HSP90AA1 \| MMP2 \| MMP9 \| TNF \| \| --- \| --- \| --- \| --- \| --- \| |
| Coronavirus disease | 0.0088 | 20.55 | 6 | \| F2 \| EGFR \| IL2 \| MMP1 \| PRKCA \| \| --- \| --- \| --- \| --- \| --- \| |
| Folate biosynthesis | 0.009 | 20.45 | 3 | \| AKR1B1 \| AKR1B10 \| ALPI \| \| --- \| --- \| --- \| |
| Pentose and glucoronate interconversions | 0.015 | 18.23 | 3 | \| AKR1B1 \| AKR1B10 \| GUSB \| \| --- \| --- \| --- \| |
| Progesterone-mediated oocyte maturation | 0.019 | 17.21 | 4 | \| HSP90AA1 \| HSP90AB1 \| PGR \| RPS6KA3 \| \| --- \| --- \| --- \| --- \| |
| Transcriptional misregulation in cancer | 0.021 | 16.77 | 5 | \| MET \| ELANE \| HDAC1 \| MMP9 \| PPARG \| \| --- \| --- \| --- \| --- \| --- \| |
| Serotonergic synapse | 0.026 | 15.85 | 4 | \| APP \| ALOX15 \| ALOX5 \| PRKCA \| \| --- \| --- \| --- \| --- \| |
| Steroid hormone biosynthesis | 0.045 | 13.46 | 3 | \| CYP3A4 \| HSD11B1 \| STS \| \| --- \| --- \| --- \| |
| P13-Akt signaling pathway | 0.045 | 13.46 | 6 | \| MET \| EGFR \| HSP90AA1 \| HSP90AB1 \| IL2 \| PRKCA \| \| --- \| --- \| --- \| --- \| --- \| --- \| |
| Breast cancer | 0.048 | 13.18 | 4 | \| EGFR \| ESR1 \| ESR2 \| PGR \| \| --- \| --- \| --- \| --- \| |
